# Protective effect of endogenous hydrogen sulfide against oxidative stress in gastric ischemia-reperfusion injury

**DOI:** 10.3892/etm.2012.870

**Published:** 2012-12-20

**Authors:** JIE CUI, LEI LIU, JIHE ZOU, WEILI QIAO, HONG LIU, YOUJIAN QI, CHANGDONG YAN

**Affiliations:** 1 Department of Physiology, Xuzhou Medical College, Xuzhou 221004, P.R. China; 2 Department of Pathology, Xuzhou Medical College, Xuzhou 221004, P.R. China

**Keywords:** gastric ischemia-reperfusion injury, hydrogen sulfide, oxidative stress, DL-propargylglycine

## Abstract

Hydrogen sulfide (H_2_S) is a gaseous signaling molecule, which plays a critical role in a number of physiological and pathological progresses. In order to determine the effect of endogenous H_2_S on gastric ischemia-reperfusion (GI-R), we evaluated the gastric mucosal damage in rats intraperitoneally injected with DL-propargylglycine (PAG, 50 mg/kg/day) or L-cysteine (L-cys, 50 mg/kg/day) for 7 days before GI-R. GI-R injury was achieved by clamping the celiac artery for 30 min, followed by reperfusion for 60 min. Gastric mucosal damage was macroscopically assessed in the area of injury and deep damage was assessed by histopathological scoring. PAG increased the area of gastric mucosal injury and deep damage compared with that in untreated GI-R rats (P<0.05). While PAG decreased the H_2_S concentration and cystathionine γ-lyase (CSE) expression in the gastric mucosa, L-cys significantly attenuated the effects of GI-R. Western blot analysis revealed that the increases of malondialdehyde (MDA) and xanthine oxidase (XOD), and decreases of glutathione (GSH), superoxide dismutase (SOD) and the restriction of superoxide (O_2_^−^) production in the PAG group were inhibited by L-cys (P<0.05). Endogenous H_2_S has a protective effect against GI-R in rats by inhibiting oxygen free radical overproduction.

## Introduction

Reperfusion, the prompt restoration of the blood supply to the ischemic tissue, is the most effective way to reduce the process of ischemic injury, which ultimately leads to cell death. However, reperfusion following even brief periods of ischemia causes irreversible damage, known as ischemia-reperfusion injury (1). Although the mechanisms underlying ischemia-reperfusion injury are complicated, oxidative stress is considered to play a pivotal role.

Hydrogen sulfide (H_2_S) was the third gaseous signaling molecule to be discovered, following nitric oxide and carbon monoxide (2). H_2_S is produced endogenously from cysteine by the pyridoxal-5′-phosphate-dependent enzymes, cystathionine β-synthase (CBS) and/or cystathionine γ-lyase (CSE). Previously, a number of studies have suggested that H_2_S has anti-inflammatory, anti-oxidative and anti-apoptotic effects (3,4). H_2_S inhibits lipid peroxidation during heart ischemia-reperfusion and decreases the mortality of myocardial cells induced by ischemia by reducing oxygen free radicals (4). Also, in brain ischemia-reperfusion injury, H_2_S exerts a protective effect on neurons by eliminating oxygen free radicals (5). Studies have shown that H_2_S has protective effects on the heart, brain, liver and lung in the case of ischemia-reperfusion injury (6–9). In the gastrointestinal tract, it has been reported that the systemic administration of sodium hydrogen sulfide (NaHS), a H_2_S donor, attenuates gastric mucosal injury by downregulating mRNA expression and plasma release of proinflammatory cytokines in rats (10). The protective effect of exogenously administered H_2_S and its precursors NaHS and L-cysteine (L-cys), has been shown against ischemia-reperfusion injury. However, the potential of endogenous H_2_S has not yet been investigated. The aim of this study was to investigate whether endogenous H_2_S plays a role in gastric ischemia-reperfusion (GI-R) injury.

We hypothesize that H_2_S plays an important role in the gastric mucosa under ischemia-reperfusion. Thus, we explored the effect of endogenous H_2_S on rat GI-R injury by pretreatment with DL-propargylglycine (PAG) and L-cys to block CSE and H_2_S synthetase and provide a precursor of H_2_S synthesis. Furthermore, we determined whether the effect of endogenous H_2_S is related to oxidative enzymes involved in GI-R.

## Materials and methods

### Animals

Adult male Sprague-Dawley rats weighing 200–250 g were provided by the Animal Department of Xuzhou Medical College. All animal care and experimental protocols complied with the Animal Management Rules of the Ministry of Health of the People’s Republic of China and the guidelines for the Care and Use of Laboratory Animals of Xuzhou Medical College.

### Chemicals

L-cys and PAG were purchased from Sigma-Aldrich (St. Louis, MO, USA). Malondialdehyde (MDA), glutathione (GSH), superoxide dismutase (SOD) and superoxide anion (O_2_^−^) assay kits were purchased from Nanjing Jiancheng Bioengineering Institute (Nanjing, China). Rabbit polyclonal antibodies to SOD-1 (sc-11407) and xanthine oxidase (XOD, sc-20991) were purchased from Santa Cruz Biotechnology, Inc. (Santa Cruz, CA, USA). Rabbit polyclonal antibodies to CSE (BA2198) were purchased from Wuhan Boster Biological Technology Co. Ltd., (Wuhan, China). Goat anti-rabbit IgG was purchased from Beijing Zhong Shan-Golden Bridge Biological Technology Co., Ltd. (Beijing, China). All other chemicals and reagents were of analytical grade.

### GI-R injury model

The rats were fasted without water deprivation for 24 h before the experiments. After inducing anesthesia with an intraperitoneal injection of 10% chloral hydrate (400 mg/kg body weight), the rats were fixed on an operating table. Following laparotomy, the celiac artery was carefully separated from surrounding tissues, clamped with an artery clamp to induce ischemia and later removed to allow reperfusion (11). Following surgery, all rats were sacrificed under anesthesia and the stomach was carefully excised for determination of gastric mucosal damage or stored at −80°C for MDA and GSH assays, and the measurement of XOD and SOD expression by western blot analysis.

### Measurement of H_2_S concentration in gastric mucosa and serum

Following sacrifice, a blood sample was rapidly collected from each rat and centrifuged at 4,000 rpm for 4 min. Then the supernatant was used for H_2_S measurement using a commercially-available kit (12). The mucosa of the stomach was scraped off, homogenized and centrifuged and then the supernatant was collected for the measurement of H_2_S concentration.

### Measurement of gastric mucosal damage

The stomach was cut open along the greater gastric curvature, rinsed and flattened. The general injury area of the gastric mucosa was calculated using Adobe Photoshop 6.0 (Adobe Systems Inc., San Jose, CA, USA). The injury area was expressed as the percentage of congestion, edema and erosion in the whole gastric mucosa area. A 1-mm tissue sample was removed from between the greater and lesser gastric curvatures and fixed in 10% formaldehyde, then processed by routine paraffin embedding, sectioning and hematoxylin and eosin (H&E) staining. The degree of pathological injury was assessed under a light microscope (each sample was blindly evaluated by a pathologist). The degree of injury was scored according to the Masuda criteria (13) with slight modification: normal, 0; injury in surface epithelium, 1; congestion and edema in the upper mucosa, 2; congestion, hemorrhage and edema in the middle and lower mucosa, 3; structural disorder or necrosis in the upper mucosal glands, 4 and deep necrosis and ulceration, 5. The average injury score for each section was calculated. The mean of 10 visual scores from each slide was calculated as the score for one rat.

### Assay of MDA and GSH content, SOD activity and the inhibition of O_2_^−^ production

The gastric mucosa was made into a 10% tissue homogenate and centrifuged at 3,000 rpm, 4°C for 10 min; then the supernatant was collected. Following the manufacturer’s instructions, the MDA and GSH contents in the supernatant were assessed by the thiobarbituric acid and 3,3′-dithiobis(6-nitrobenzoic) acid methods, respectively. The protein content was determined by the bicinchoninic acid (BCA) method. SOD activity in the supernatant was evaluated by the inhibition of XOD, according to the manufacturer’s instructions and expressed as units per milligram tissue (U/mg). Inhibition of O_2_^−^ production was measured according to the manufacturer’s instructions and expressed as units per gram tissue (U/g).

### Western blot analysis of CSE, XOD and SOD

Gastric mucosal protein was extracted according to the BCA method. The concentration of each sample was diluted to the same level, then mixed with 1/3 volume protein denaturation solution and boiled for 5 min to denature the protein. Samples were separated on 10% sodium dodecyl sulphate (SDS)-polyacrylamide gels. Membranes were probed with polyclonal antibodies to CSE, SOD and XOD overnight at 4°C. The secondary antibody was conjugated to horseradish peroxidase with a BCIP/NBT kit (Promega Corporation, Madison, WI, USA). β-actin was used to normalize for loading variations.

### Experimental protocol

The rats were randomly divided into 4 groups with 10 per group: i) sham group, age-matched healthy rats were only laparotomized without celiac artery clamping; ii) GI-R group, age-matched healthy rats were intraperitoneally injected with normal saline for 7 days, then the celiac arteries were clamped for 30 min ischemia and then reperfused for 60 min; iii) PAG group, the rats were intraperitoneally injected with the H_2_S synthetase blocker PAG (50 mg/kg/day) for 7 days, then the celiac arteries were clamped for 30 min ischemia and then reperfused for 60 min and iv) L-cys group, the rats were intraperitoneally injected with L-cys (50 mg/kg/day) for 7 consecutive days, then the celiac arteries were clamped for 30 min ischemia and then reperfused for 60 min.

### Statistical analysis

All data are expressed as mean ± standard deviation (SD). Statistical analysis was performed by SPSS 13.0 (SPSS Inc., Chicago, IL, USA) for Windows. Statistical significance was calculated by one-way analysis of variance. P<0.05 was considered to indicate a statistically significant difference.

## Results

### Gastric mucosal injury

**Figure 1.**
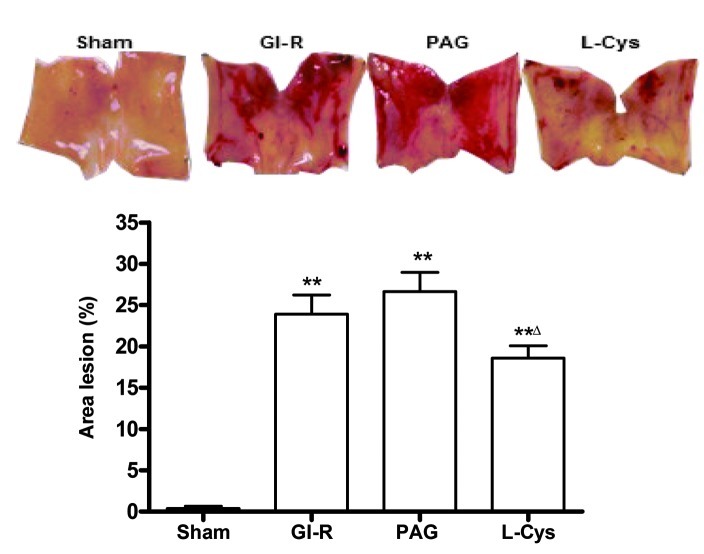
Effects of PAG and L-cys on gastric mucosal injury area in rats subjected to gastric ischemia-reperfusion (GI-R). Male Sprague-Dawley rats were intraperitoneally injected with the H_2_S synthetase blocker PAG (50 mg/kg) or L-cys (50 mg/kg) for 7 days before the celiac arteries were clamped for 30 min ischemia and then reperfused for 60 min. Sham group, age-matched rats with physiological solute treatment but no GI-R procedure; GI-R group, age-matched rats with physiological solute treatment followed by the GI-R procedure; PAG group, rats intraperitoneally injected with PAG and then subjected to the GI-R procedure; L-cys group, rats intraperitoneally injected with L-cys and then subjected to the GI-R procedure. Data presented as mean ± standard deviation (SD); n=8. ^**^P<0.01 vs. the sham group; ^Δ^P<0.05 vs. the PAG group. PAG, DL-propargylglycine; L-cys, L-cysteine; H_2_S, hydrogen sulfide.

**Figure 2.**
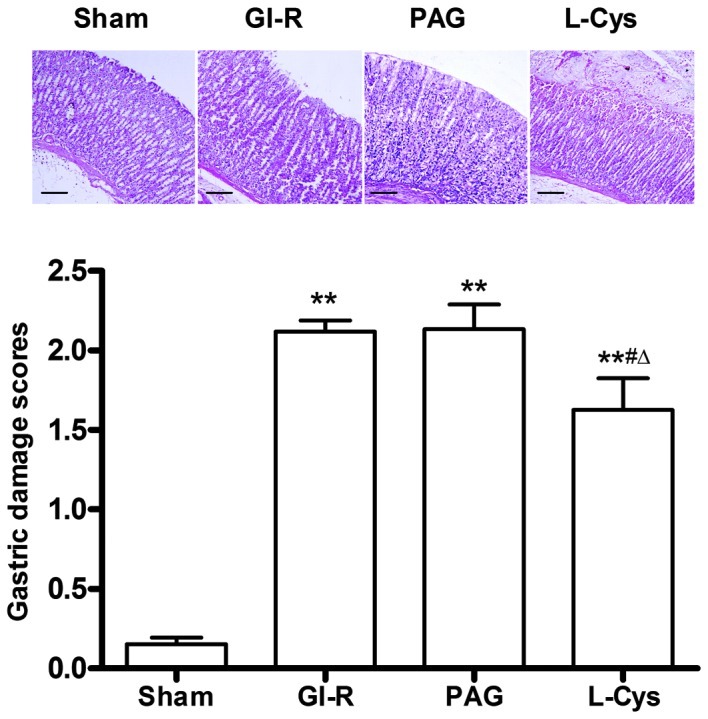
Effects of PAG and L-cys on gastric mucosal injury scores in rats subjected to gastric ischemia-reperfusion (GI-R) (hematoxylin and eosin stain; magnification, ×100). Male Sprague-Dawley rats were intraperitoneally injected with the H_2_S synthetase blocker PAG (50 mg/kg) or L-cys (50 mg/kg) for 7 days before the celiac arteries were clamped for 30 min ischemia and then reperfused for 60 min. Sham group, age-matched rats with physiological solute treatment but no GI-R procedure; GI-R group, age-matched rats with physiological solute treatment followed by the GI-R procedure; PAG group, rats intraperitoneally injected with PAG and then subjected to the GI-R procedure; L-cys group, rats intraperitoneally injected with L-cys and then subjected to the GI-R procedure. Scale bar, 200 μm. Data presented as mean ± standard deviation (SD), n=8. ^**^P<0.01 vs. the sham group, ^#^P<0.05 vs. the GI-R group, ^Δ^P<0.05 vs. the PAG group. PAG, DL-propargylglycine; L-cys, L-cysteine; H_2_S, hydrogen sulfide.

The mucosal surface of the sham group was smooth and no significant abnormality in the gastric mucosa was observed under the light microscope. However, significant hemorrhage and edema and several erosions of varying depths and sizes were observed on the surface of the mucosa of the GI-R group. The epithelial cells and gland ducts of the mucosa at the hemorrhage sites were shed and disorganized in the GI-R group. Compared with the GI-R group, the injury area and the extent of mucosal damage significantly increased in the PAG group and L-cys inhibited the damage induced by GI-R (P<0.05; Fig. 1). Under the light microscope, the gastric damage scores in the GI-R, PAG and L-cys groups were much higher than those in the sham group (P<0.01). However, the damage score in the L-cys group was lower than those of the GI-R and PAG groups (P<0.05, Fig. 2).

### H_2_S concentration in the serum and gastric mucosal tissue

**Figure 3.**
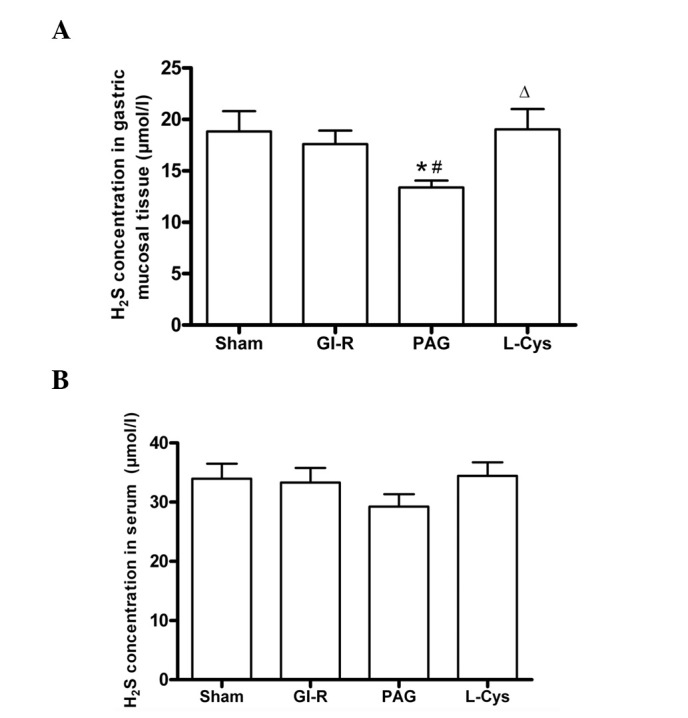
Effects of PAG and L-cys on H_2_S concentration in the gastric mucosa (A) and serum (B) from rats subjected to gastric ischemia-reperfusion (GI-R). Male Sprague-Dawley rats were intraperitoneally injected with the H_2_S synthetase blocker PAG (50 mg/kg) or L-cys (50 mg/kg) for 7 days before the celiac arteries were clamped for 30 min ischemia and then reperfused for 60 min. Sham group, age-matched rats with physiological solute treatment but no GI-R procedure; GI-R group, age-matched rats with physiological solute treatment followed by the GI-R procedure; PAG group, rats intraperitoneally injected with PAG and then subjected to the GI-R procedure; L-cys group, rats intraperitoneally injected with L-cys and then subjected to the GI-R procedure. Data presented as mean ± standard deviation (SD), n=8. ^*^P<0.05 vs. the sham group, ^#^P<0.05 vs. the GI-R group, ^Δ^P<0.05 vs. the PAG group. PAG, DL-propargylglycine; L-cys, L-cysteine; H_2_S, hydrogen sulfide.

Although there were no significant changes in H_2_S concentration in the serum (Fig. 3B), compared with the sham and GI-R groups, PAG decreased the concentration of H_2_S in the gastric mucosa (P<0.05) and L-cys attenuated this decrease (P<0.05). However, there was no significant difference in H_2_S level in the gastric mucosa between the GI-R and sham groups (Fig. 3A).

### CSE expression in the gastric mucosa

**Figure 4.**
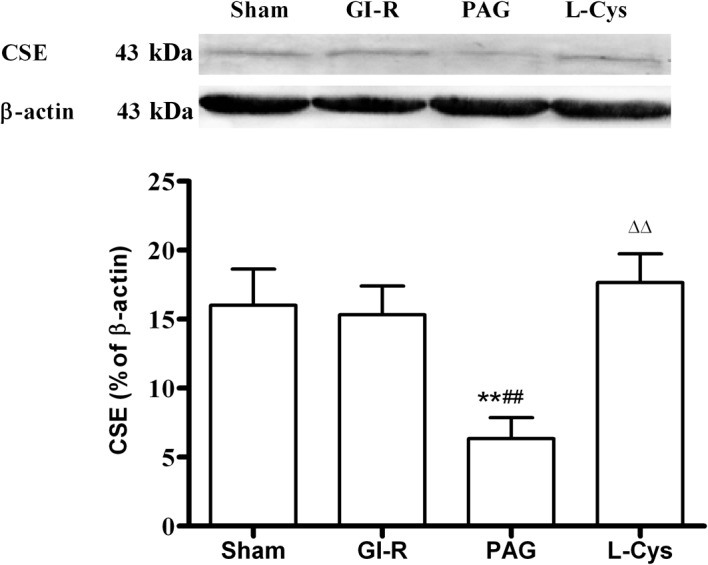
Effects of PAG and L-cys on CSE expression in gastric mucosa from rats subjected to gastric ischemia-reperfusion (GI-R). Male Sprague-Dawley rats were intraperitoneally injected with the H_2_S synthetase blocker PAG (50 mg/kg) or L-cys (50 mg/kg) for 7 days before the celiac arteries were clamped for 30 min ischemia and then reperfused for 60 min. Sham group, age-matched rats with physiological solute treatment but no GI-R procedure; GI-R group, age-matched rats with physiological solute treatment followed by the GI-R procedure; PAG group, rats intraperitoneally injected with PAG and then subjected to the GI-R procedure; L-cys group, rats intraperitoneally injected with L-cys and then subjected to the GI-R procedure. Data presented as mean ± standard deviation (SD), n=3. ^**^P<0.01 vs. the sham group, ^##^P<0.01 vs. the GI-R group, ^ΔΔ^P<0.01 vs. the PAG group. PAG, DL-propargylglycine; L-cys, L-cysteine; H_2_S, hydrogen sulfide; CSE, cystathionine γ-lyase.

Compared with the sham group, GI-R alone had no effect on CSE expression. PAG significantly inhibited the expression of CSE (P<0.01) and L-cys increased CSE in the gastric mucosa to normal levels (P<0.01; Fig. 4).

### MDA and GSH contents in the gastric mucosa

**Figure 5.**
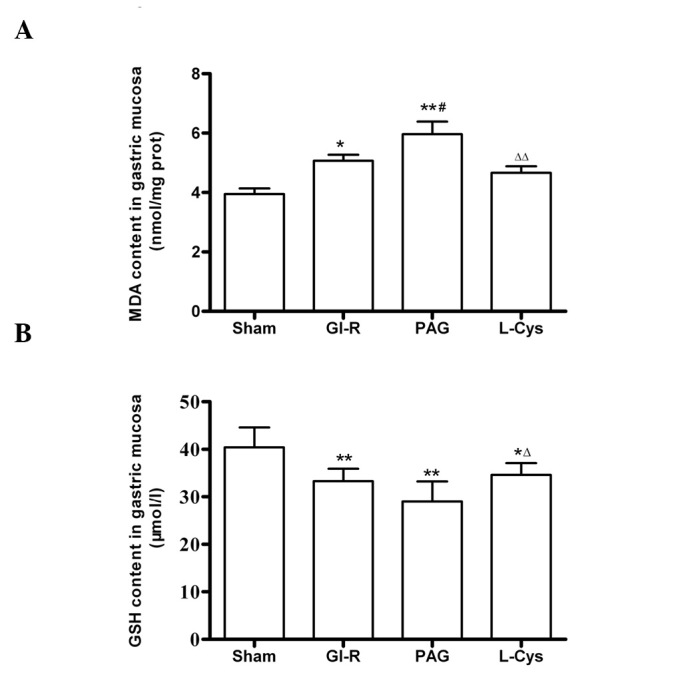
Effects of PAG and L-cys on (A) MDA and (B) GSH content in gastric mucosa from rats subjected to gastric ischemia-reperfusion (GIR). Male Sprague-Dawley rats were intraperitoneally injected with the H_2_S synthetase blocker PAG (50 mg/kg) or L-cys (50 mg/kg) for 7 days before the celiac arteries were clamped for 30 min ischemia and then reperfused for 60 min. Sham group, age-matched rats with physiological solute treatment but no GI-R procedure; GI-R group, age-matched rats with physiological solute treatment followed by the GI-R procedure; PAG group, rats intraperitoneally injected with PAG and then subjected to the GI-R procedure; L-cys group, rats intraperitoneally injected with L-cys and then subjected to the GI-R procedure. Data presented as mean ± standard deviation (SD), n=8. ^*^P<0.05, ^**^P<0.01 vs. the sham group; ^#^P<0.05 vs. the GI-R group; ^Δ^P<0.05, ^ΔΔ^P<0.01 vs. the PAG group. PAG, DL-propargylglycine; L-cys, L-cysteine; H_2_S, hydrogen sulfide; MDA, malondialdehyde; GSH, glutathione.

The MDA content of the mucosa increased following GI-R (P<0.05) and further increased in the PAG group. However, L-cys decreased the concentration of MDA in mucosa (Fig. 5A). The GSH content in the mucosa decreased in the GI-R, PAG and L-cys groups. However, the reduction of GSH content in the L-cys group was less than that in the PAG group (P<0.05; Fig. 5B).

### XOD and SOD expression, SOD activity and inhibition of O_2_^−^ production in the gastric mucosa

**Figure 6.**
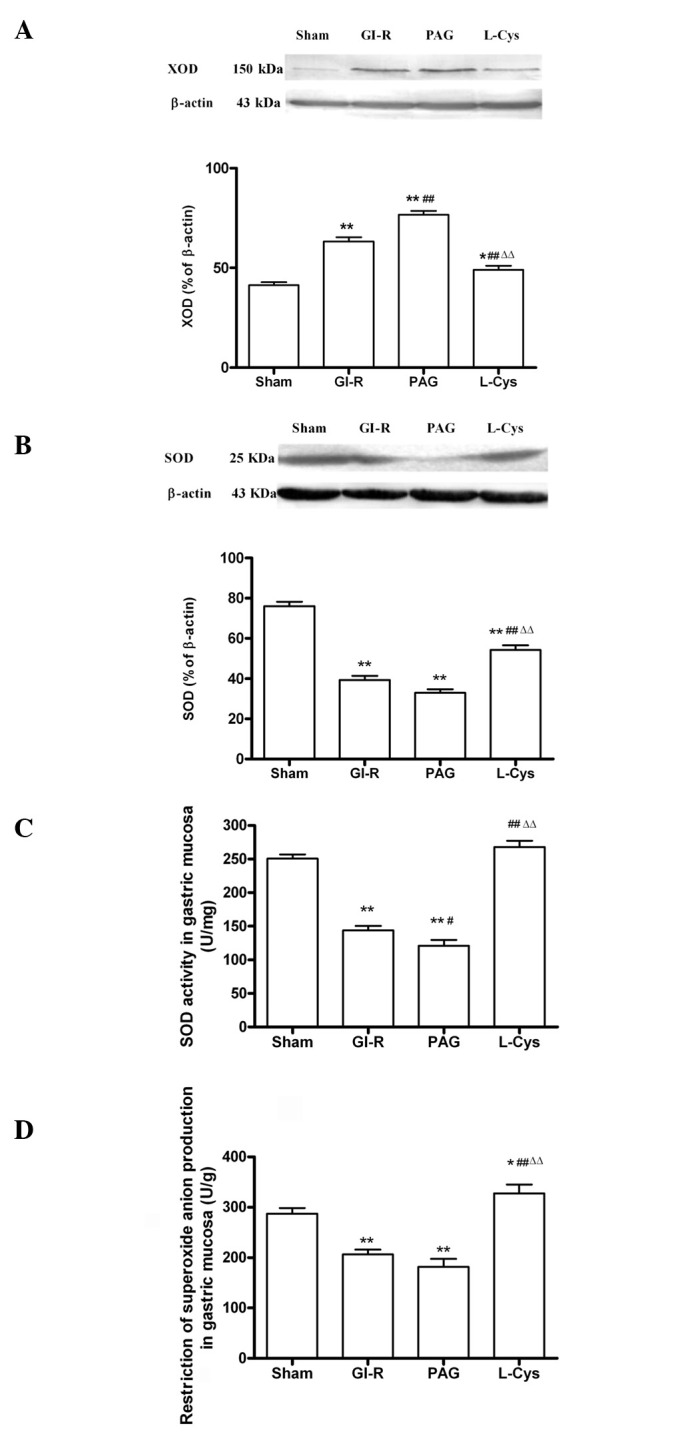
Alteration of oxidative stress in gastric mucosal tissue from rats subjected to 30 min gastric ischemia and 60 min reperfusion (GI-R). (A) Effects of PAG and L-cys on XOD expression in gastric mucosal tissue induced by GI-R (n=3). (B) Effects of PAG and L-cys on SOD expression in gastric mucosal tissue induced by GI-R (n=3). β-actin was used to normalize for loading variations. (C) Effects of PAG and L-cys on SOD activity in gastric mucosal tissue induced by GI-R (n=8). (D) Effects of PAG and L-cys on the alteration of O_2_^−^ production in gastric mucosal tissue induced by GI-R. Male Sprague-Dawley rats (n=8) were intraperitoneally injected with the H_2_S synthetase blocker PAG (50 mg/kg) or L-cys (50 mg/kg) for 7 days before the celiac arteries were clamped for 30 min ischemia and then reperfused for 60 min. Sham group, age-matched rats with physiological solute treatment but no GI-R procedure; GI-R group, age-matched rats with physiological solute treatment followed by the GI-R procedure; PAG group, rats intraperitoneally injected with PAG and then subjected to the GI-R procedure; L-cys group, rats intraperitoneally injected with L-cys and then subjected to the GI-R procedure. Data presented as mean ± standard deviation (SD). ^*^P<0.05, ^**^P<0.01 vs. the sham group, ^#^P<0.05, ^##^P<0.01 vs. the GI-R group, ^ΔΔ^P<0.01 vs. the PAG group. PAG, DL-propargylglycine; L-cys, L-cysteine; H_2_S, hydrogen sulfide; XOD, xanthine oxidase; SOD, superoxide dismutase.

Compared with the sham group (P<0.01; Fig. 6A), the expression level of XOD markedly increased in the GI-R and PAG groups; while the SOD expression in the GI-R and PAG groups decreased compared with the sham group (P<0.01; Fig. 6B). Compared with the GI-R group, L-cys downregulated XOD (P<0.01) and upregulated SOD (P<0.01). Additionally, the activity of SOD was decreased in the GI-R and PAG groups and the effects of GI-R were attenuated by L-cys (P<0.01; Fig. 6C), as was the inhibition of O_2_^−^ production (Fig. 6D).

## Discussion

The occurrence of GI-R injury is related to a number of factors, including excessive production of oxygen free radicals in the mucosa (14), leukocyte infiltration (15) and decreased release of nitric oxide (16). Oxidative stress induced by high levels of active oxygen plays a major role in GI-R injury. Excess production of oxygen free radicals is a major initiating factor and an independent pathogenic factor of GI-R injury (17,18). Therefore, investigating how to decrease oxidative stress is essential for developing means of protecting the gastric mucosa from attack by deleterious factors.

H_2_S is well-known as a toxic gas with the smell of rotten eggs. Nevertheless, with the increasing interest in endogenous gaseous signaling molecules, it has been shown that endogenous H_2_S regulates a range of physiological and pathological processes in the nervous, digestive and cardiovascular systems (19–22). Hence, H_2_S is considered to be a physiologically important molecule and a gaseous mediator. In mammals, endogenous H_2_S is generated from L-cys under the catalysis of total CBS and CSE, 1/3 of which is in the form of gas while 2/3 is in the form of NaHS. NaHS dissociates *in vivo* into sodium ions and sulfhydryl group ions and the latter bind with hydrogen ions to generate H_2_S. Thus, H_2_S and NaHS are in dynamic equilibrium (17). L-cys administration is cardioprotective through enhanced myocardial H_2_S generation as a result of CSE activation, which is attenuated by the selective CSE enzyme inhibitor, PAG (23). Meanwhile, H_2_S protects brain endothelial cells from oxidative stress (24). In this study, we identified that in rats pretreated with a H_2_S synthetase blocker (PAG, 50 mg/kg/day) for 7 days, significant hemorrhage, edema and erosions were observed in the surface of the gastric mucosa, as well as in the GI-R group. However, pretreatment with L-cys, the precursor of H_2_S, protected the mucosa from the damage induced by GI-R (Fig. 1). The same results were observed for gastric damage scores. After blocking the production of H_2_S with PAG, gastric mucosal ulceration and the number of hemorrhage sites significantly increased, a number of mucosal cells died, erosion sites formed and significant edema and inflammatory infiltration appeared in the submucosa. The signs of gastric mucosal injury were markedly alleviated by the continuous administration of L-cys (Fig. 2). Although there was no significant change in the H_2_S concentration in serum, PAG successfully inhibited H_2_S production (Fig. 3) and CSE expression (Fig. 4) in the gastric mucosa, while L-cys did not cause any evident change in H_2_S. Endogenous H_2_S in the gastric mucosa plays an important role in the protective effect against GI-R injury. Whiteman *et al* identified that in the rat brain, H_2_S protects neurons from injury by eliminating oxygen free radicals (5).

A number of studies have reported that oxidative stress plays a pivotal role in GI-R injury. In the GI-R process, lipid peroxidation is induced by an increased number of oxygen free radicals. A change of MDA content is a measure of the degree of damage caused by membrane lipid peroxidation. In our study, when H_2_S was inhibited by PAG, lipid peroxidation increased in the gastric mucosa since the MDA content was higher than that of the GI-R group. However, when H_2_S was increased, the MDA level decreased (Fig. 5A). This result is consistent with the findings on the effect of H_2_S on a rat model of myocardial infarction (25). Furthermore, GSH is the major endogenous antioxidant produced by mammalian cells, preventing damage to important cellular components caused by reactive oxygen species (26). We identified that L-cys increased the GSH content when compared with the PAG group (Fig. 5B). When H_2_S production was blocked, MDA levels increased. Additionally, increasing endogenous H_2_S production reduced MDA content since GSH levels were enhanced. This suggests that endogenous H_2_S protects the integrity of the gastric mucosa by increasing GSH and decreasing MDA content. This is consistent with the finding that H_2_S has anti-oxidative effects by promoting the transfer of cystine into cells and improving the cellular synthesis of GSH (27). The antioxidant action of H_2_S plays an important role during GI-R.

During the reperfusion period following ischemia or hypoxia, large amounts of superoxide and O_2_^−^ are generated from xanthine and hypoxanthine under the action of XOD. XOD is considered the most important source of oxygen free radicals (28). Meanwhile, living tissues are endowed with innate antioxidant defense mechanisms, namely antioxidative enzymes. When the amount of active oxygen exceeds the scavenging capacity of the antioxidant defensive system, including SOD, the immunological function of the gastrointestinal tract is severely damaged by free radicals, which results in tissue and organ injury (29). In this study, we identified that blocking the synthesis of endogenous H_2_S significantly increased the expression of XOD (Fig. 6A), causing oxygen free radical overproduction in the gastric mucosa. Nevertheless, the activity of the antioxidative enzyme, SOD, was enhanced by increasing H_2_S in rats pretreated with L-cys (Fig. 6B and C). As a result, the ability to inhibit O_2_^−^ production was clearly reduced due to the reduction of endogenous H_2_S by PAG (Fig. 6D). The protective role of H_2_S against oxidative stress has also been clarified in rat gastric mucosal epithelium (30).

In conclusion, this study demonstrates that endogenous H_2_S protects the gastric mucosa against ischemia and reperfusion injury. Selective inhibition of the CSE enzyme enhances the injury following GI-R. However, L-cys administration attenuates the harmful effects of GI-R through activation of CSE to increase H_2_S generation. The gastroprotective effect of endogenous H_2_S against GI-R may be mediated by enhancing the anti-oxidative capacity through increasing GSH and SOD to reduce free radical production.

## Abbreviations:

H_2_S: hydrogen sulfide;
GI-R: gastric ischemiareperfusion;
PAG: DL-propargylglycine;
MDA: malondialdehyde;
GSH: glutathione;
SOD: superoxide dismutase;
CSE: cystathionine γ-lyase;
XOD: xanthine oxidase;
L-cys: L-cysteine
